# Impact of Peracetic Acid on the Dynamic Cyclic Fatigue of Heat-Treated Nickel-Titanium Rotary Endodontic Instrument

**DOI:** 10.1155/2021/6676005

**Published:** 2021-01-19

**Authors:** Suhad Jabbar Hamed Al-Nasrawi, Zuha Ayad Jaber, Nibrass Talib Al-Quraine, Abtesam Imhemed Aljdaimi, Sattar Jabbar Abdul-Zahra Al-Hmedat, Saleh Zidan, Julfikar Haider

**Affiliations:** ^1^Department of Conservative Dentistry, Faculty of Dentistry, University of Kufa, Najaf, Iraq; ^2^College of Dentistry and Oral Surgery, Alasmarya of Dental Materials, Faculty of Dentistry, Sebha University, Sebha, Libya; ^3^Department of Dental Materials, Faculty of Dentistry, Sebha University, Sebha, Libya; ^4^Department of Engineering, Manchester Metropolitan University, Manchester M1 5GD, UK

## Abstract

Peracetic acid (PAA) is widely used as a sterilizing/disinfecting agent, and, in endodontics, it has been introduced as a promising irrigant in root canal treatment. It has been used at different concentrations to achieve various functions. However, endodontic instruments in contact with PAA of a certain concentration may affect their fatigue resistance. Therefore, the aim of this study was to investigate the impact of PAA on the cyclic fatigue resistance of three commercial heat-treated nickel-titanium (NiTi) rotary files. Three types of heat-treated NiTi rotary files were selected: One Curve (OC), ProTaper Gold (PTG), and Wave One Gold (WOG). Each type was divided into three subgroups (*n* = 6 for each file type): (1) untreated instruments; (2) files immersed in 0.002% PAA; and (3) files immersed in 0.35% PAA. The performance of each file type was tested in a simulated canal. The number of cycles to fracture (NCF) was determined to assess cyclic fatigue resistance of the files. Independent sample *t*-test was applied to compare each treated file within a subgroup with its respective control group, and one-way ANOVA was used for comparison among the main groups. All types of tested files revealed a significant decline in the cyclic fatigue resistance after exposure to 0.002% PAA except the PTG (*P*=0.209). After exposure of the files to a higher concentration (0.35% PAA), a dramatic reduction was demonstrated by all the groups. Before and after exposure of the files to PAA, PTG displayed the highest cyclic fatigue resistance, followed by the WOG, while the OC showed the lowest resistance. Exposure of heated-treated NiTi files to PAA in a relatively high or low concentration adversely affects the cyclic fatigue resistance. The PTG files demonstrated the best performance among the tested types and can be disinfected with 0.002% PAA for clinical purpose.

## 1. Introduction

Peracetic acid (PAA) or peroxyacetic acid is a mixture of hydrogen peroxide and acetic acid in an aqueous solution. It is a well-known oxidative and bleaching agent, commonly used as a disinfectant and sanitizer for contaminated surfaces, processing of plants, and equipment [1, 2]. It possesses a broad spectrum of activity against Gram-positive and Gram-negative bacteria, fungi, yeasts, and viruses. It acts rapidly and efficiently to remove organic material, without leaving any residues. One of the interesting advantages of its usage is the low toxicity of its decomposition products (acetic acid, hydrogen peroxide, water, and oxygen). In addition, it remains effective even in the existence of organic materials, suspended solids at low temperatures, with long term application potential. Nevertheless its application has been restricted because of its corrosive properties [3]. In dentistry, it has been recommended for sterilization and disinfection of equipment [4], impression [5], and gutta-percha [6]. Moreover, it has been introduced as a promising intracanal irrigant with antibacterial [7] and tissue dissolving properties [8].

In relation to sterilization, it has been used in diverse schemes of various concentrations, contact times, and temperatures to achieve the function of sterilization or disinfection. Spores of *Bacillus atrophaeus*, the most resistant ones to chemical disinfection [9], were demonstrated to be effectively eradicated with the aid of 0.2% PAA after 40 min contact time [10]. The virucidal efficacy against *Human rotavirus* could be achieved with 0.014% of PAA at 30 min exposure. *Echovirus* 1, *Poliovirus* 1, *Coxsackievirus B5*, *Human rotavirus*, and *Bacteriophage f2 r*equired lower concentration for inactivation. On the other hand, *Simian rotavirus* SA11 that was the most sensitive one needed just 0.002% for inactivation [11]. At a low concentration of PAA, Harakeh [12] demonstrated that the acid at up to 0.0007% was an effective bacteriocide but not a good virucide. However, other researchers stated that the minimum virus inactivation dosage is 12 ppm (0.0012%) [13]. However, for the destruction of viruses (HBV, HIV) and vegetative bacteria, only 5 min is required, while the destruction of sporicidal function requires 10 min immersion in 0.35% PAA [14]. Ossia-Ongagna and Sabatier [15] supported the use of PAA at 0.35% with 10-minute contact period to achieve the sporicidal activity.

In dentistry, disinfection of alginate impressions by immersing in 0.2% peracetic acid provided a comparable effect to that by 2% glutaraldehyde, 2.5% sodium hypochlorite (NaOCl), and chlorhexidine (CHX) [5]. In orthodontics, PAA at 0.25% for 10 min was suggested for decontamination of orthodontic plier [4]. Furthermore, it provided rapid decontamination of the gutta-percha [6]. A 0.2% peracetic acid also offered a high level of disinfection for polymerized acrylic resins contaminated with *Bacillus subtilis* after 5 min contact [16]. In an experiment to investigate the sterilizing capacity and a possible adverse effect of usage of PAA on dental equipment, Ceretta et al. [17] demonstrated the efficient and safe sterilization of stainless steel dental equipment immersed in 0.25% of PAA for 20 min, while causing minimal corrosive damage.

The root canal treatment is a multiphase treatment where various instruments are involved to achieve good cleaning and shaping of the root canal and removing of bacterial inhabitants. Although some of the intracanal instruments have been considered as single-use instruments, an instrument like endodontic file, as packaged by the manufacturer, is not sterile and should, therefore, be sterilized before the first use. Moreover, the packages carry a clear label stating that the contents are not sterilized [18]. Additionally, their surfaces might be a site of contamination with metallic spurs and debris [19]. In some conditions, epithelial cells have been observed on new or unused files [20]. Therefore, preuse sterilization appears to be an essential requirement where the PAA is one of the commonly suggested materials.

Effective root canal instrumentation and debridement are greatly affected by combined use of an irrigant such as NaOCl, ethylenediaminetetraacetate, and hydrogen peroxide, which has great impact on the performance of the instrument [21]. Many studies have been conducted to improve the cleaning and disinfection of the root canal system using PAA. Peracetic acid at 1% revealed an effective bactericidal effect against *Enterococcus faecalis*, providing a comparable effect to that of 2.5% NaOCl and 2% CHX [22]. Another study stated that mixed biofilms could be significantly eradicated and dissolved using 4% PAA [7]. Moreover, at 5%, PAA could dissolve pulp tissue equally effectively as 2.5% and 5.25% NaOCl [8]. Its antibacterial effectiveness accompanied by the capability to eliminate the smear layer has made the PAA a promising irrigant, which would speed up and simplify the preparation of root canal [23]. In an investigation into the combined use of 0.05% PAA with rotary instruments, Gomes et al. [24] introduced it as an alternative to 2.5% NaOCl for removing the smear layer. However, exposure of the intracanal instrument to a chemical agent during irrigation or chemical sterilization/disinfection could cause surface alterations and corrosion which adversely affect the cutting efficiency of the blades [25], overall strength [26], and fracture resistance [27] of the endodontic files.

In the modern dental clinics, rotary endodontic instruments, particularly those made of NiTi alloys, are commonly employed in root canal treatment. The NiTi instruments possess several favorable properties such as corrosion resistance and superelasticity with excellent shape memory that enable them to easily navigate through the intricate root canal systems to achieve the required cleaning and shaping [28, 29]. In spite of these favorable properties, fatigue and failure result in possible intracanal fracture of the instrument.

Flexural and torsional stresses are the most noteworthy factors which could cause the unexpected file breakage [30]. During canal instrumentation, the torsional fracture could take place following bending of the tip or a part of the file inside the root canal while the remaining part still rotates, overloading the torsional resistance of the file [31]. In contrast, repetitive file compression and tension could cause cyclic fatigue failures [32]. Cyclic fatigue stresses result in the formation of microcracks on the file's surface, usually beginning with minimal defects on the instrument outer part [33]. File materials, design, geometrical discontinuities, inclusions, porosities, and overheating that occurred during manufacturing could lead to fatigue failure [34]. Furthermore, environmental factors such as temperature and type of irrigation solutions might affect the file strength [21].

Therefore, it is important that the instrument has sufficient resistance against fatigue when it is in contact with a sterilizing agent. As the PAA has been applied as a sterilizing agent and an intracanal irrigant, its effect on the heat-treated NiTi rotary instrument at high and low concentrations is worth investigating. However, there is no information in the literature on the impact of peracetic acid on the cyclic fatigue resistance of the endodontic instrument. Thus, the aim of this study was to assess the influence of peracetic acid at two concentrations (0.002% and 0.35%) on the cyclic fatigue resistance of the endodontic files.

The tested null hypotheses in the current study were as follows: (1) there would be no significant effect on the cyclic fatigue resistance of the three types of heat-treated NiTi rotary endodontic files in contact with lower concentration of PAA (0.002 wt.%); (2) there would be no significant effect on the cyclic fatigue resistance of the same three types of files in contact with higher concentration of PAA (0.35 wt.%).

## 2. Materials and Methods

### 2.1. File Samples and Preparation

Fifty-four heat-treated NiTi rotary endodontic files were selected for the fatigue resistance test. All the instruments were of comparable size and taper (#25, 0.06). The files were initially observed under stereomicroscope (Meiji Techno Co. Ltd., Tokyo, Japan) at 20x magnification in order to ensure that they were defect free.  Table 1 presents further details about different file groups.

Each group had 18 unused files that were allocated into three subgroups (*n* = 6). The sample size was decided after running a pilot study on pair of files from each group. In the first subgroup, the files were left untreated to act as a control subgroup. Each file in the second subgroup was immersed in 0.002% PAA (Bonnymans, Willowyard Industrial Estate, UK) for 10 min at 35°C, while each file in the third subgroup was immersed in 0.35% PAA for 10 min at 35°C. The PAA solutions are freshly prepared from 5% stock solution. After the immersion, the files were thoroughly rinsed with distilled water and dried to get rid of any effect for the PAA.

### 2.2. Cyclic Fatigue Testing

**Figure 1:**
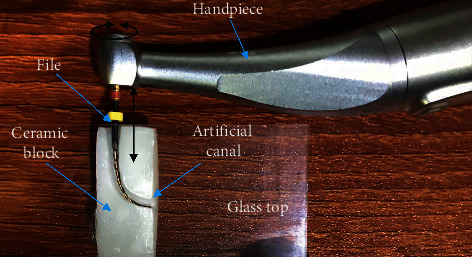
Zirconia block with artificial canal for cyclic fatigue testing of endodontic files.

Files were examined for cyclic fatigue resistance using an artificial canal at 35 ± 1°C [35]. The artificial canal was created inside a block of zirconia with 0.6 mm apical diameter, 6.06 mm radius, and 45° curvature angle. The canal curvature started approximately 2.5 mm from the canal tip, and the maximum curvature was about 5.5 mm from the tip (Figure 1). It was prepared on a presintered zirconia block, and then the block was sintered in a sintering oven (Vita Zyrcomat, Vita Zahnfabrik, Germany) for 1 hour at 1500°C to obtain the full strength of the canal [36].

To avoid slipping out and to allow good observation of the instruments during testing, the simulated canal was covered with a glass plate. According to the manufacturer's recommendation, the WOG files were used with a reciprocal mode of rotation (clockwise + anticlockwise), while the OC and PTG files underwent full clockwise rotation. The rotation was performed with a constant speed of 350 rpm utilizing a contra-angle and motorized by a torque-controlled electric motor (X-Smart, Dentsply Maillefer, Ballaigues, Switzerland) in back-and-forth axial movements till the files fracture. The axial movements (dynamic) for all files were 3 mm, with nearly 2 sec for every displacement to simulate the actual clinical situation. That was achieved with the aid of circular lines on the file shaft as they were separated by 1 mm. The dynamic movement in each cycle consisted of in and out steps: the file was first inserted to the full working length, moved outward by 3 mm (3 lines), and then moved inward to full working length again. Following each file change, the artificial canal was irrigated with normal saline to reduce the friction between the instrument and the canal walls.

The time-to-fracture was counted with a 1/100 s chronometer. The experimental part of this study was carried out by an expert dentist, with the help of an assistant for time recording. The number of cycles to failure (NCF) was defined by transferring the time needed to fracture into decimal units and then multiplying the time by the stated number of rotations per minute. The NCF data (*n* = 54) obtained from nine subgroups were averaged to compute the mean NCF value for each subgroup. The lengths of the resulting fractured fragments were measured using a digital caliper (Mitutoyo, Kawasaki, Japan).

### 2.3. Fractographic Analysis

The cross-sectional and lateral views of the fractured files were examined under a scanning electron microscope (Carl Zeiss Ltd, 40 VP, Smart SEM, Cambridge, UK) at various magnifications (×200 to ×5,000) to observe the fracture behaviors. The fatigue signs were manifested in four areas: [1] crack initiation, where microcracks form and start to grow; [2] crack propagation, where the crack continuously grows; [3] overstressed area or overload zone (a typical dimple rupture or ductile failure) [4, 37, 38] and signs of edge wear with rounding of cross-sectional outline and loss of sharpness of the cutting edges.

### 2.4. Statistical Analysis

For each subgroup, the mean and standard deviation of the NCF was recorded. The Shapiro–Wilk test for normality indicated that the data of the NCF were normally distributed, and therefore the independent samples *t*-test was performed to define any statistically significant differences between the subgroups before and after the acid exposure at the low and high concentrations. After that, one-way ANOVA test was implemented to find differences between the main groups before acid exposure, after exposure to the low concentration, and after exposure to the high concentration. Bonferroni test followed the ANOVA test to specify the significance between the main groups. As the data of the fragment lengths were not normally distributed, nonparametric tests were used to compare the results. Mann–Whitney *U* test was applied to compare the fractured fragment lengths in the experimental subgroups with that of the control, while Kruskal–Wallis test was applied to compare the data of the main groups. SPSS software version 22.0 (IBM Corp., Armonk, USA) was used for the statistical analysis, and all tests were conducted at a confidence level of 95%, and *P* < 0.05.

## 3. Results

**Figure 2:**
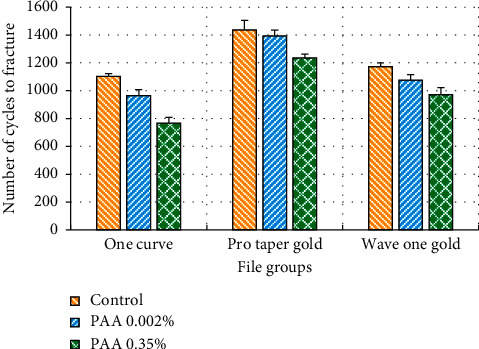
Mean and standard deviation values of cyclic fatigue resistance for the tested file groups.

As illustrated in Table 2 and Figure 2, PTG showed the greatest cyclic fatigue resistance (1437.33 ± 67.59), while the OC files were the lowest (1103.67 ± 18.96). All of the tested groups showed a reduction in the cyclic fatigue resistance after the exposure to 0.002% PAA. The reduction was significant, except for the PTG group (*P*=0.209). After the file exposure to 0.35% PAA, all of the tested groups showed a greater significant reduction in the cyclic fatigue resistance. The largest reduction was demonstrated by the OC (by 12.53% and 30.44% at 0.002% and 0.35% concentrations of PAA, respectively), followed by the WOG (by 8.37% and 17.10% at 0.002% and 0.35%, respectively) and then PTG (by 2.99% and 13.96% at 0.002% and 0.35%, respectively).

Before acid exposure, the PTG group revealed the highest fatigue resistance, while the OC group showed the lowest fatigue resistance. They demonstrated the same trend after acid exposure at both concentrations. From the comparison between the main groups, the differences between untreated OC and WOG groups were significant (*P*=0.04), while the differences between the other groups in all the sets were highly significant (Table 3).

Table 4 shows the mean and standard deviation of the fractured fragment lengths for all groups. Without any PAA exposure, the OC recorded the shortest fragment length, while WOG was the longest. The differences between the tested groups were significant except those between the PTG and WOG (*P*=0.130) as shown in Table 5. However, after acid exposure at 0.002% or 0.35% PAA, the differences were not statistically significant except those between the OC and WOG.

**Figure 3:**
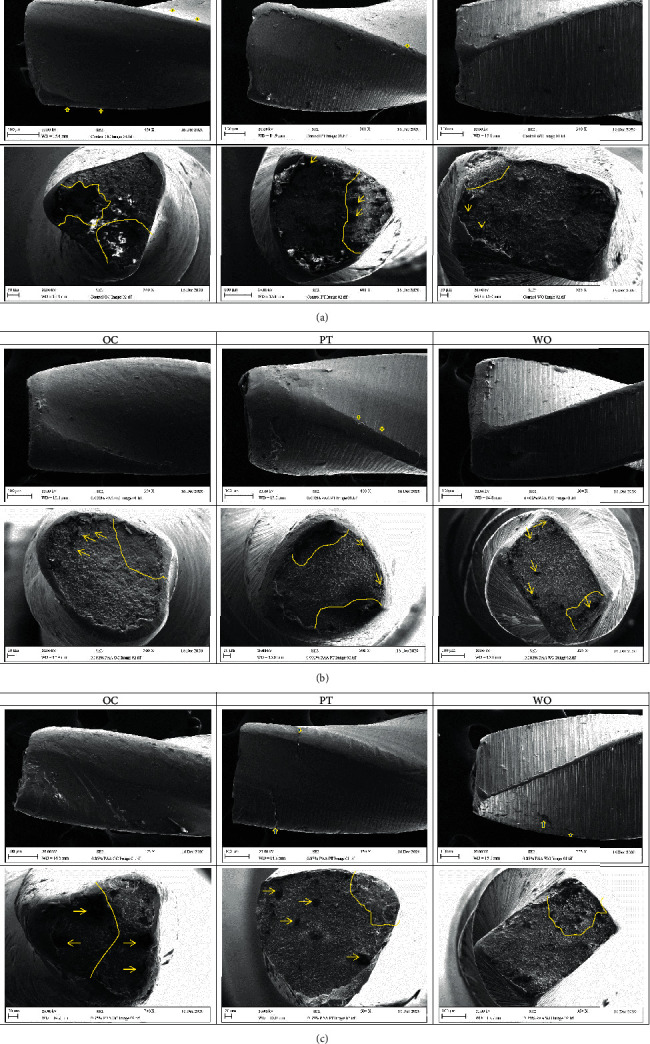
Scanning electron micrographs of failed files: (a) control subgroups, (b) 0.002% PAA subgroups, (c) 0.35% PAA subgroups showing the lateral view of the fractured files having numerous cracks near the fracture sites (thick arrows), with surface wear (erosion) of the cutting edges represented by short straight lines. The cross-sectional views of crack initiation areas (dotted line) and surface pitting with voids (thin arrow).

As seen in Figure 3, the scanning electron microscopic analysis near the fractured surfaces of the control subgroups showed microcracking (thick arrows) particularly in the OC, with irregular fracture line in all tested files. However, the PTG and WOG files immersed in 0.35% PAA showed even stronger evidence of microcracking. In addition, sign of metal wear at the file cutting edges was identified in the groups treated with 0.002% PAA. The wear progressively increased after immersion in 0.35% PAA, particularly in the OC and PTG. The cross-sectional views revealed features of mechanical damage in all groups. The crack initiation areas (dotted line) and the overloaded fast fracture zones with voids, pitting (thin arrows), could be identified. In comparison to the control subgroups, the cross-sectional outlines of the acid treated subgroups became more rounded. The OC showed an aggressive sign of failure with larger number of bigger dimples.

## 4. Discussion

Endodontic files unexpectedly fail at the file's maximum flexure point due to the cyclic fatigue while rotating inside the curved root canals [39]. Advanced NiTi instrument might come into contact with PAA during the file sterilization or canal irrigation. Thus, it is highly important to carry out cyclic fatigue tests in order to provide the dentists with information about the possible effect of PAA on the file resistance to fracture. This investigation compares the cyclic fatigue of three types of heat-treated NiTi rotary endodontic files before and after 10-minute immersion in a relatively low (0.002%) or high (0.35%) concentration of PAA. The 0.35% concentration was selected because of its efficient rapid sterilization efficacy [14], while the 0.002% represented an effective disinfectant at low concentration [11]. However, PAA was used in endodontics as high as 5% [8], so the result of this study draws the attention of researchers to the adverse effect of this acid on the files.

This study basically was designed to test the effect of the PAA exposure on the cyclic fatigue resistance of different types of files. In order to maintain consistency, the files in the subgroups of each main group were standardized as they belonged to same type of files, where the result after each treatment was compared to the control group of the same type using independent sample *t*-test. The effect of file design, alloy used, and motion type on file response to PAA exposure is worth investigating. This was demonstrated by comparing the response of the commercially available files with different types of designs, materials, and operational instructions. In the current study, the three selected types of heat-treated NiTi files of similar size and taper were tested under standardized experimental conditions. As PTG was similar to WOG in terms of heat treatment during manufacturing [40], the comparison between them reflected the effect of the file mode of motion and the cross-sectional design. On the other hand, both PTG and OC had triangular cross section and underwent continuous clockwise rotation, but they were made from different alloys. Therefore, a comparison between them reflected the effect of file materials on the cyclic fatigue resistance.

According to the outcomes of this study, treatment with 0.002% or 0.35% PAA had a significant influence on the file resistance to cyclic fatigue except for PTG type with 0.002% PAA. Therefore, the first hypothesis was partially rejected. As the exposure to 0.35% PAA significantly reduced the cyclic fatigue for all the tested types of files, the second null hypothesis was totally rejected.

Cyclic fatigue results of the nonimmersed files revealed that the PTG files displayed the highest fatigue resistance, followed by the WOG, while the OC files showed the lowest resistance. This trend in result was in agreement with a study by Abdul-Zahra Al Hmedat on WOG and OC files without any application of PAA [36]. The findings of Yilmaz et al. [41] also supported the present result as they stated that OC files showed lower cyclic fatigue resistance than that of the WOG files. As for the comparison between the OC and PTG, the current result also agreed with Uygun et al. [42] as they reported that PTG files demonstrated higher cyclic fatigue resistance than OC instruments. On the other hand, the current result disagreed with Hanbazaza and Abuhaimed [43] as they demonstrated that PTG with continuous rotation failed at lower fatigue cycles than the WOG with the reciprocal rotation. This disagreement can be assumed due to the difference in experimental set-up employed.

The major factors affecting cyclic fatigue resistance of the files include file size, taper, cross-sectional design, manufacturing techniques, and materials [44, 45]. During cyclic fatigue test, the point of maximum stress could influence the fatigue life of NiTi rotary instruments. The fatigue resistance is reduced with large metal volume [46]. Generally, there are similarities between the PTG and WOG as they were manufactured through complicated heating–cooling proprietary treatment, were subjected to gold treatment technology [40], and possessed constant tapers between D1 and D3, which decreased progressively from D4 to D14. However, there were also differences between the two files. A convex triangular cross-sectional design in PTG was different from a parallelogram-shaped design with two cutting edges in WOG [40]. The design of file cross section proved to be responsible for altering the distribution of stress in a rotary instrument under twisting or tension [47, 48]. A finite elemental analysis proved that higher resistance to cyclic fatigue was exhibited by the triangular cross-sectional design than that shown by a square cross-sectional design of file [47]. This was attributed to the lesser metal mass of the file with a triangular cross section compared to the one with a square cross section and of a similar diameter [49, 50]. Hence, in this study, the higher resistance to cyclic fatigue files might be due to the smaller metal mass of the triangular cross section in PTG compared to additional metal mass in the parallelogram-shaped cross section of the WOG file. This finding agreed with those of previous studies reporting that the files manufactured from thermally treated alloy and small cross section exhibit greater resistance to cyclic fatigue [50–52]. Moreover, the cross-sectional design might affect the engagement of file blade with the canal wall and stress generation, being also advantageous in reducing torsional stresses [53]. It was stated that the amount of metal mass could affect the fatigue resistance of rotary instruments [54]. In relation to the file kinetics, although researches demonstrated that reciprocal motion could enhance the file fatigue resistance [43], the effect of cross-sectional design appears to be more influential as demonstrated in the current study. It was stated that less flexibility, less stress distribution along the length, and highest stress concentration are related to the rotary system with rectangular cross section in comparison with a triangular and S-shaped cross section. Therefore, the rectangular cross section design is more susceptible to plastic deformation [53, 54].

Both of the PTG and WOG files demonstrated better cyclic fatigue resistance than OC. This might be due to the type of alloy and wire, file design, and method of thermal treatment employed. The OC is made of C wire [55], while PTG and WOG are made of a gold-wire with advanced metallurgy and a two-phase transformation heat-treated alloy, which involves two main steps: initial electropolishing and subsequent heat treatment [56]. Furthermore, OC has a variable asymmetrical off-centered cross section of a convex triangular tip with S-shaped coronal section [57]. After the file exposure to the PAA, the files displayed the same trend of performance, with PTG being the best, followed by WOG, while the OC displayed the lowest value of cyclic fatigue resistance. To date, no studies on the effect of PAA on heat-treated rotary NiTi instruments have been published yet. Immersion of the files in 0.002% PAA caused dropping of the values of cyclic fatigue resistance in all types of tested files. The PTG files remained the best and nonsignificantly affected by the low PAA concentration. However, the higher concentration of PAA (0.35%) dramatically reduced the fatigue resistance of all types, and the PTG remained the best of all types, followed by the WOG and OC. This could be attributed to the corrosive effect of the PAA on the materials the file was made of. Thierry et al. [58] also stated that PAA resulted in a scattered breakdown potential in the immersed NiTi alloy.

In relation to the effect of acid, the current results agree with that of Pulikkottil et al. [59] as they reported that the corrosion resistance of NiTi wires had reduced significantly after exposure to an acidic solution [41]. Regarding the effect of acid on mechanical property of the NiTi alloy, the result of the current study is in harmony with Lin et al. [26] findings, as they demonstrated that, at an acidic pH, the three-point bending strength of NiTi wire significantly decreased. It was reported that, with the increase of concentration of an acid, the pH decreased causing stronger corrosive effect [60]. Therefore, the significant decrease in cyclic fatigue resistance of the files could be attributed to the accelerating corrosive effect at high acid concentration.

Regardless of the treatment, interestingly, the fracture site in the OC file was located near the beginning of the canal curvature, while for the PTG and WOG, it was near the center of the canal curvature. Local stress concentration in the file determines the location of crack initiation and subsequent fracture [61], which may occur owing to microscopic defects or microcracks [62]. The current result was in agreement with Uygun et al. [42] as they reported that the fragment length of PTG group (5.34 ± 0.53 mm) was greater than that of the OC group (4.54 ± 0.2 mm). In the present study, the fragment length of OC (2.57 ± 0.087 mm) was also less than that of the PTG (4.06 ± 1.09 mm) without any treatment. On the other hand, the present finding disagreed with that of Yilmaz et al. [41] as the WOG and OC had comparable fragment length. Again, the fragment length results (Table 4) after both the acid treatments were not quite consistent. However, these differences require further research to identify the influencing factors. Transformational design of OC cross section [63] might modify the distribution of the maximum stress points [64]. The difference in fragment lengths among the groups could be attributed to the effect of the test condition, the properties of the alloy used, or the original internal defects within the files.

Scanning electron micrographs of the control subgroups showed signs of mechanical failure as microcracking, crack propagation, and overloaded zones. While the acid treated subgroups showed further wear signs such as flattened edge and rounded cross section with large pitting related to the acid immersion. These signs were clear in the OC confirming the adverse effect of the PAA immersion on the cyclic fatigue of the tested files.

A significant laboratory drawback in investigating the cyclic fatigue resistance of NiTi rotary files could be multiple contributing variables towards fatigue failure, such as study environment, material properties, specific design, and dimensions of the tested instrument. All that made it difficult to measure the influence of a single variable on file resistance to cyclic fatigue [47]. The clinical relevance of the outcomes of these laboratory studies was difficult to evaluate due to the experimental conditions that differed from the clinical intracanal instrumentation, where the file failure might occur due to the factors (cyclic fatigue and torsional stress) that acted simultaneously [65]. Besides, Yao et al. [66] demonstrated that although the use of extracted teeth simulated clinical situations, they were not anatomically standardized. Thus, the extracted teeth were not optimal for examining the cyclic fatigue resistance for NiTi files. In this study, an artificial canal was utilized to rule out all variables other than the cyclic fatigue variable.

The cyclic fatigue resistance of rotary NiTi files has been studied by rotating the NiTi files against an inclined plane utilizing a grooved block or a curved metal tube [67, 68]. The performance test could be conducted in either dynamic or static mode. In the static mode, the instrument rotates in the artificial canal at constant length without any axial movement [69]. However, in the dynamic mode, the rotation of the file in the artificial canal was associated with an axial movement. Dynamic modes were recorded to be more realistic in the simulation of clinical situations than the modes with no axial movement [70]; therefore, the dynamic model was applied in the current study. In addition, the reciprocal motion employed in WOG files compared to the other two could bring some degree of inconsistency in the results. Moreover, in order to provide such simulation, the current study was conducted at 35°C, which is the reported intracanal temperature. However, it was stated that the NiTi files might be affected by the intracanal temperature [71].

Further chemical analysis is required to investigate the effect of the PAA on the chemical composition of the tested files possibly due to corrosion. In addition, fracture behavior of the samples can be investigated to identify the failure modes and mechanisms in the file types.

For the root canal treatment with commercially available rotary endodontic files, it is recommended that sterilizing agent (PAA) should be avoided in order to avoid any adverse fatigue failure.

## 5. Conclusions

Within the limitations of the present in vitro study, it can be concluded that the use of PAA sterilizing agent at a relatively high (0.35%) or low (0.002%) concentration adversely affects the cyclic fatigue resistance of the heated-treated NiTi rotary files used for root canal treatment. The PTG files were the best among the tested types (OC and WOG) and could be disinfected or used with 0.002% PAA without any significant loss of fatigue resistance. However, high concentration of PAA for sterilization/disinfection or as an irrigation solution in conjunction with heat-treated NiTi rotary files is contraindicated. PTG files could be safely used for clinical root canal treatment at low concentration of PAA.

## Figures and Tables

**Table 1:** Instrument groupings and their characteristics.

Group number	File groups	Cross-sectional design	Material	Manufacturing	Operational procedure
Group 1	One Curve (OC; Micro-Mega, Besancon, France)	Variables from triangular to S shapes at apical to coronal part	NiTi: C wire	Heat-treated	Dynamic-continuous

Group 2	ProTaper Gold (PTG); Dentsply Maillefer, Ballaigues, Switzerland)	Convex triangular	NiTi: gold-wire	Heat-treated	Dynamic-continuous

Group 3	Wave One Gold (WOG; Dentsply Sirona, Ballaigues, Switzerland)	Parallelogram	NiTi: gold-wire	Heat-treated	Dynamic-reciprocal (clockwise 150° and anticlockwise 30°)

**Table 2:** The means and standard deviations (SD) of the number of cycles to failure of the tested files.

File treatment	Fatigue resistance of file groups		
	One Curve	ProTaper Gold	Wave One Gold
Control (no PAA)	1103.67 (18.96)	1437.33 (67.59)	1173.67 (25.93)
0.002% PAA	965.42 (41.69)^*∗*^	1394.25 (40.09)	1075.49 (39.69)^*∗*^
0.35% PAA	767.75 (37.59)^*∗*^	1236.67 (26.09)^*∗*^	973.00 (48.86)^*∗*^

^*∗*^A statistically significant difference in the independent samples *t*-test.

**Table 3:** *P* values of multiple comparisons by ANOVA/Bonferroni test for NCF before and after immersion in 0.002% or 0.35% PAA.

File treatment	Groups		*P* value^*∗*^
Control (no PAA)	One Curve	ProTaper Gold	≤0.001
		Wave One Gold	≤0.040
	ProTaper Gold	Wave One Gold	≤0.001

0.002 wt.% PAA	One Curve	ProTaper Gold	≤0.001
		Wave One Gold	≤0.001
	ProTaper Gold	Wave One Gold	≤0.001

0.35 wt.% PAA	One Curve	ProTaper Gold	≤0.001
		Wave One Gold	≤0.001
	ProTaper Gold	Wave One Gold	≤0.001

^*∗*^Significant when *P* value ≤0.05.

**Table 4:** The means and standard deviations (SD) of the fragmented lengths of the tested files.

File treatment	Fragment lengths in the file groups (mm)		
	One Curve	ProTaper Gold	Wave One Gold
Control (no PAA)	2.57 (0.087)	4.06 (1.09)	5.53 (0.64)
0.002 wt.% PAA	2.765 (0.36)^*∗*^	3.68 (0.55)^*∗*^	5.58 (0.12)^*∗*^
0.34 wt.% PAA	2.566 (0.25)^*∗*^	3.92 (1.13)^*∗*^	6.31 (0.41)^*∗*^

^*∗*^A statistically significant difference (*P* < 0.05) in the Mann–Whitney *U* test.

**Table 5:** *P* values of multiple comparisons by Kruskal–Wallis tests for the fragmented lengths of the tested file groups.

File treatment	Groups		*P* value
Control (no PAA)	One Curve	ProTaper Gold	0.030
		Wave One Gold	0.000
	ProTaper Gold	Wave One Gold	0.130

0.002% PAA	One Curve	ProTaper Gold	0.197
		Wave One Gold	0.000
	ProTaper Gold	Wave One Gold	0.135

0.35% PAA	One Curve	ProTaper Gold	0.251
		Wave One Gold	0.000
	ProTaper Gold	Wave One Gold	0.120

## Data Availability

The data used to support the findings of this study are available from the corresponding author. Requests for access to these data should be made to Suhad Al-Nasrawi (suhad.alnasrawi@uokufa.edu.iq).
